# Parent-Reported History of Traumatic Dental Injuries and Current Sports Participation Among Paediatric Dental Patients in Central Poland: A Cross-Sectional Study

**DOI:** 10.3390/jcm15197720

**Published:** 2026-10-07

**Authors:** Karolina Spodzieja, Dorota Olczak-Kowalczyk

**Affiliations:** Department of Paediatric Dentistry, Medical University of Warsaw, 02-097 Warsaw, Poland; karolina.spodzieja@wum.edu.pl

**Keywords:** traumatic dental injuries, dental trauma, paediatric dentistry, sports participation, epidemiology, children, Poland

## Abstract

**Background:** Traumatic dental injuries (TDIs) are among the most common oral health problems in children and adolescents and may significantly affect quality of life and long-term oral health outcomes. Participation in sports activities has been identified as an important risk factor for dental trauma; however, epidemiological data concerning Polish paediatric populations remain limited. **Objective:** The aim of the present study was to assess the proportion and characteristics of parent-reported traumatic dental injuries among children attending a university paediatric dental clinic in the Mazowieckie Voivodeship and to evaluate the associations between a parent-reported history of dental trauma, current sports participation, and selected demographic characteristics. **Materials and Methods:** A cross-sectional questionnaire-based study was conducted among parents and legal guardians of children aged 7–15 years attending the Department of Paediatric Dentistry at the Medical University of Warsaw. Participants were recruited using convenience sampling during routine dental visits between 2023 and 2025. The questionnaire included items concerning demographic characteristics, current sports participation, and the child’s history of traumatic dental injuries. Statistical analyses included chi-square tests, Fisher’s exact tests, Mann–Whitney U tests, and logistic regression analysis. **Results:** A total of 735 questionnaires were included in the analysis. Traumatic dental injuries were reported in 42.9% of children. A history of TDI was reported for 47.7% of boys and 37.3% of girls (*p* = 0.006). A reported history of dental trauma was more common among children currently participating in sports than among non-participants (49.7% vs. 31.3%; *p* < 0.001). Football participation was associated with a higher occurrence of reported TDI. Rural residence and lower parental education level were also associated with a higher occurrence of reported TDI. In the fitted logistic regression model, current sports participation was associated with higher odds of a reported TDI history (OR = 2.33; 95% CI: 1.69–3.21). **Conclusions:** A history of traumatic dental injuries was frequently reported among children attending a university paediatric dental clinic in the Mazowieckie Voivodeship and was associated with sports participation and selected demographic characteristics. Owing to the clinic-based convenience sampling, these findings should not be interpreted as population-level prevalence estimates for children in Central Poland.

## 1. Introduction

Traumatic dental injuries (TDIs) constitute a significant public health problem among children and adolescents worldwide. Due to their high prevalence, potential long-term complications, and impact on quality of life, TDIs are considered one of the major oral health challenges in paediatric dentistry. Dental trauma may result in pain, aesthetic impairment, functional disturbances, psychological consequences, and substantial treatment costs, particularly when permanent teeth are affected [1,2].

The prevalence of TDIs in children varies considerably between countries and populations, depending on age, diagnostic criteria, socioeconomic conditions, and study methodology. According to a global meta-analysis conducted by Petti S and colleagues, traumatic dental injuries affect approximately 15% of permanent teeth and nearly 23% of primary teeth worldwide, with an estimated one billion people having experienced dental trauma during their lifetime [3]. Epidemiological studies have reported prevalence rates ranging from 20% to 40% among school-aged children [4,5]. In Poland, available studies also indicate a high frequency of dental trauma among paediatric populations, emphasising the need for preventive strategies and improved public awareness [6].

Children are particularly susceptible to dental trauma due to high levels of physical activity, developing motor coordination, and increasing participation in organised sports and recreational activities In a three-year retrospective study of traumatic injuries to permanent teeth at the University Dental Clinic of Vienna, fractures were the most common injury type, accounting for 53% of affected teeth [7]. Numerous studies demonstrated that boys experience traumatic dental injuries more frequently than girls. For example, Dua and colleagues reported that males accounted for 63.2% of TDI cases, compared with 36.4% among females [8]. This difference has been associated with greater participation in contact sports and more risk-taking behaviours among boys [9].

Falls are considered the most common cause of traumatic dental injuries in children. Patnana et al. demonstrated that falls were responsible for approximately 59.3% of all TDIs of primary teeth in children, while Dua et al. reported that falls during play accounted for 37.5% of injuries [8,10]. Other common causes include sports-related accidents, bicycle accidents, playground injuries, collisions, and interpersonal violence [11]. The type and aetiology of trauma often vary according to age, sex, and environmental conditions.

Participation in sports has been identified as an important risk factor for TDIs. Previous studies showed that children engaged in organised sports are significantly more likely to sustain dental trauma compared with non-athletes, especially when protective equipment such as mouthguards is not routinely used [12,13]. Sports such as football, basketball, martial arts, hockey, and cycling have been particularly associated with an increased risk of dental injuries [14]. Olczak-Kowalczyk et al. reported that approximately one in four traumatic dental injuries in Polish adolescents was related to sports activity [15].

The maxillary permanent incisors are the teeth most frequently affected by traumatic injuries because of their anterior position and increased exposure during falls and collisions. Dua et al. found that maxillary central incisors accounted for approximately 43.8% of all injured teeth [8]. The most commonly reported forms of trauma include uncomplicated crown fractures, concussion and subluxation injuries, and luxation injuries [16]. In primary dentition, luxation injuries are more prevalent because of the anatomical characteristics of supporting tissues and incomplete root formation [17].

Several demographic, socioeconomic, and anatomical factors have been associated with the occurrence of traumatic dental injuries. Predisposing occlusal characteristics include increased overjet, inadequate lip coverage, and certain forms of malocclusion, which may increase the exposure of maxillary anterior teeth to direct impact [18,19]. Moreover, parental knowledge regarding the emergency management of dental trauma remains insufficient in many populations. The prognosis of an injured tooth depends on the type and severity of the injury, the stage of root development, and the time elapsed before appropriate treatment. Delayed or absent treatment may result in complications such as pulp necrosis, infection, root resorption, disturbances in the development of permanent successors, tooth loss, and adverse aesthetic or functional outcomes. Immediate professional assessment and appropriate management, particularly in cases involving tooth displacement or avulsion, are therefore essential for improving long-term outcomes and reducing complications [20].

Despite the growing body of evidence regarding traumatic dental injuries, epidemiological data concerning TDIs among children in Central Poland remain limited. Furthermore, relatively few studies have simultaneously evaluated the prevalence of TDIs, sports participation, and demographic risk factors in Polish paediatric populations.

Therefore, the aim of the present study was to assess the proportion and characteristics of parent-reported traumatic dental injuries among school-aged children attending a university paediatric dental clinic in the Mazowieckie Voivodeship and to evaluate the associations between a parent-reported history of dental trauma, current sports participation, and selected demographic characteristics.

## 2. Materials and Methods

A cross-sectional questionnaire-based study was conducted among parents and legal guardians of school-aged children attending the Department of Paediatric Dentistry at the Medical University of Warsaw. Data were collected during routine dental appointments between 2023 and 2025.

Participants were recruited using convenience sampling from patients attending this university paediatric dental clinic. Eligible children were aged 7–15 years, attended primary school, and resided in the Mazowieckie Voivodeship. The sample included children from both urban and rural areas and from communities of different sizes. However, because participants were recruited at a single university dental centre rather than through population-based random sampling, the sample should not be considered representative of all children living in the Mazowieckie Voivodeship or Central Poland.

Parents or legal guardians of children aged 7–15 years attending primary school were eligible for participation. Inclusion criteria comprised being the caregiver of a child within the specified age range, providing informed consent, and completing the study questionnaire. Questionnaires containing more than 10% missing responses were excluded from further analysis.

Of the 786 questionnaires distributed, 735 met the predefined completeness criterion and were included in the analysis. Questionnaires with more than 10% missing responses were excluded; therefore, limited item-level missing data remained in selected variables.

Before data collection, a minimum target sample size of 384 participants was estimated using a 95% confidence level and a 5% margin of error, based on the approximate number of primary-school children residing in the Mazowieckie Voivodeship reported by Statistics Poland. The final sample included 735 participants. This calculation was used to establish a pragmatic recruitment target; because participants were selected using convenience sampling at a single clinical centre, exceeding the calculated sample size does not establish population representativeness or eliminate the possibility of selection bias.

Data were collected using a structured self-administered questionnaire developed specifically for the present study based on previously published surveys concerning traumatic dental injuries (TDIs).

The questionnaire consisted of sections addressing the following:

- Demographic characteristics of parents and children;
- History and characteristics of traumatic dental injuries;
- Sports participation outside compulsory school physical education classes and type of sport practiced by the child;
- Circumstances and management of dental trauma.

The questionnaire included both single-choice and multiple-choice questions regarding the occurrence, location, and type of traumatic dental injuries, as well as first-aid procedures undertaken following injury.

Before the main data collection, a preliminary assessment of the questionnaire was conducted. Content relevance, clarity, and clinical importance were evaluated by two experts in paediatric dentistry and public health. Cognitive pretesting was subsequently performed with 15 parents to assess the comprehensibility of the questions and response options. All expert ratings were positive, resulting in I-CVI and S-CVI/Ave values of 1.00 for relevance, clarity, and clinical importance. The mean clarity rating obtained during cognitive pretesting was 3.23 on a four-point scale. Owing to the small number of experts and participants, this process was considered a preliminary content assessment and cognitive pretest rather than a comprehensive psychometric validation.

Questionnaires were distributed to parents and legal guardians during routine dental visits at the Department of Paediatric Dentistry. Participation in the study was voluntary and anonymous. Respondents completed the questionnaires independently on-site, while trained research staff remained available to provide clarification when necessary.

All collected data were anonymized before statistical analysis to ensure participant confidentiality.

The primary outcome was the proportion of children with a parent-reported history of traumatic dental injury and the reported characteristics of those injuries. Additional outcomes included the assessment of associations between TDIs, demographic variables, and sports participation.

Statistical analyses were performed using IBM SPSS Statistics version 25. Descriptive statistics were calculated for all variables. Continuous variables were expressed as means and standard deviations (SD), whereas categorical variables were presented as frequencies and percentages.

Associations between categorical variables were evaluated using the chi-square (χ^2^) test or Fisher’s exact test when appropriate. Mann–Whitney U tests were applied for comparisons involving ordinal variables. Logistic regression analysis was additionally performed to evaluate variables associated with a reported history of traumatic dental injuries. A *p*-value below 0.05 was considered statistically significant.

The study protocol was approved by the Bioethics Committee of the Medical University of Warsaw (approval no. AKBE/122/2022). All procedures were conducted in accordance with the ethical principles of the Declaration of Helsinki. Informed consent was obtained from all participants prior to enrolment in the study.

## 3. Results

### 3.1. Study Population

A total of 735 questionnaires were included in the final analysis. The study group comprised 735 children aged 7–15 years, with a mean age of 10.60 ± 2.38 years. Among them, 335 were girls (45.6%), and 398 were boys (54.1%); sex data were missing for two children (0.3%). Most participants were aged 7–12 years (74.7%), whereas 25.3% were aged 13–15 years.

The majority of questionnaires were completed by mothers or female legal guardians (62.2%). More than half of respondents had higher education (57.3%), while 36.6% reported secondary education and 5.6% primary education. Participants attending the clinic resided in both rural and urban areas, including villages and cities of different population sizes. The demographic characteristics of the study population are presented in Table 1.

### 3.2. Sports Participation

Regular participation in sports activities was reported for 62.4% of children, while 37.4% did not engage in organised sports. The most frequently practiced disciplines were swimming (15.9%), football (15.0%), martial arts (12.2%), and acrobatics/gymnastics/dance (8.6%). Other reported sports included volleyball, basketball, cycling, athletics, tennis/table tennis, horseback riding, handball, and gym training. The characteristics of current sports participation among the children are presented in Table 2.

### 3.3. Reported History and Characteristics of Traumatic Dental Injuries

A history of traumatic dental injury was reported for 315 children, corresponding to 42.9% of the study sample. Most injured children experienced dental trauma only once (68.9%), whereas repeated injuries were reported in 29.2% of cases.

Primary teeth were affected in 57.8% of children with a history of trauma. Among permanent teeth, maxillary incisors were the most commonly injured teeth (31.4%), followed by mandibular incisors (7.0%).

The most frequent location of injury was the home environment (50.5%), followed by playgrounds or sports fields (36.2%) and schools (18.1%). Street-related injuries accounted for 10.5% of traumatic events.

Regarding injury type, crown fractures were the most frequently reported injuries (34.9%), followed by concussion or subluxation injuries (29.2%). Intrusive luxation was reported in 10.2% of cases, while root fractures and tooth avulsion accounted for 7.6% and 7.3% of injuries, respectively.

Approximately half of respondents (50.5%) reported that first aid had been provided within several hours after injury. Subsequently, 59.4% of children visited a dentist following trauma, and 60.6% attended regular dental follow-up appointments after the injury. The reported characteristics, circumstances, and management of traumatic dental injuries are summarised in Table 3.

The association between traumatic dental injuries and demographic characteristics of children and their parents was analysed using χ^2^ tests for sex and age group comparisons, while Mann–Whitney U tests were applied for parental education level and place of residence.

A statistically significant association was observed between sex and a reported history of traumatic dental injuries. The proportion of children with a reported TDI history was higher among boys than among girls (47.7% vs. 37.3%; χ^2^ = 8.07, *p* = 0.006). However, the observed effect size, assessed using Cramer’s V coefficient, was low.

No statistically significant differences were found between age groups and the occurrence of traumatic dental injuries (*p* = 0.732).

Parental education level was significantly associated with a reported history of TDI. The proportion of children with a reported TDI history was highest among those whose parents had primary education (61.0%) and lowest among those whose parents had higher education (36.3%) (*p* < 0.001). Similarly, traumatic dental injuries occurred more frequently among children living in rural areas and smaller places of residence compared with children from larger urban areas (*p* = 0.013). The effect sizes for both parental education and place of residence, assessed using the r coefficient, were low.

Overall, male sex, lower parental education level, and rural residence were associated with a higher occurrence of parent-reported TDI history in the study sample. The associations between a reported history of traumatic dental injuries and the demographic characteristics of the children and their parents are presented in Table 4.

**Figure 1:**
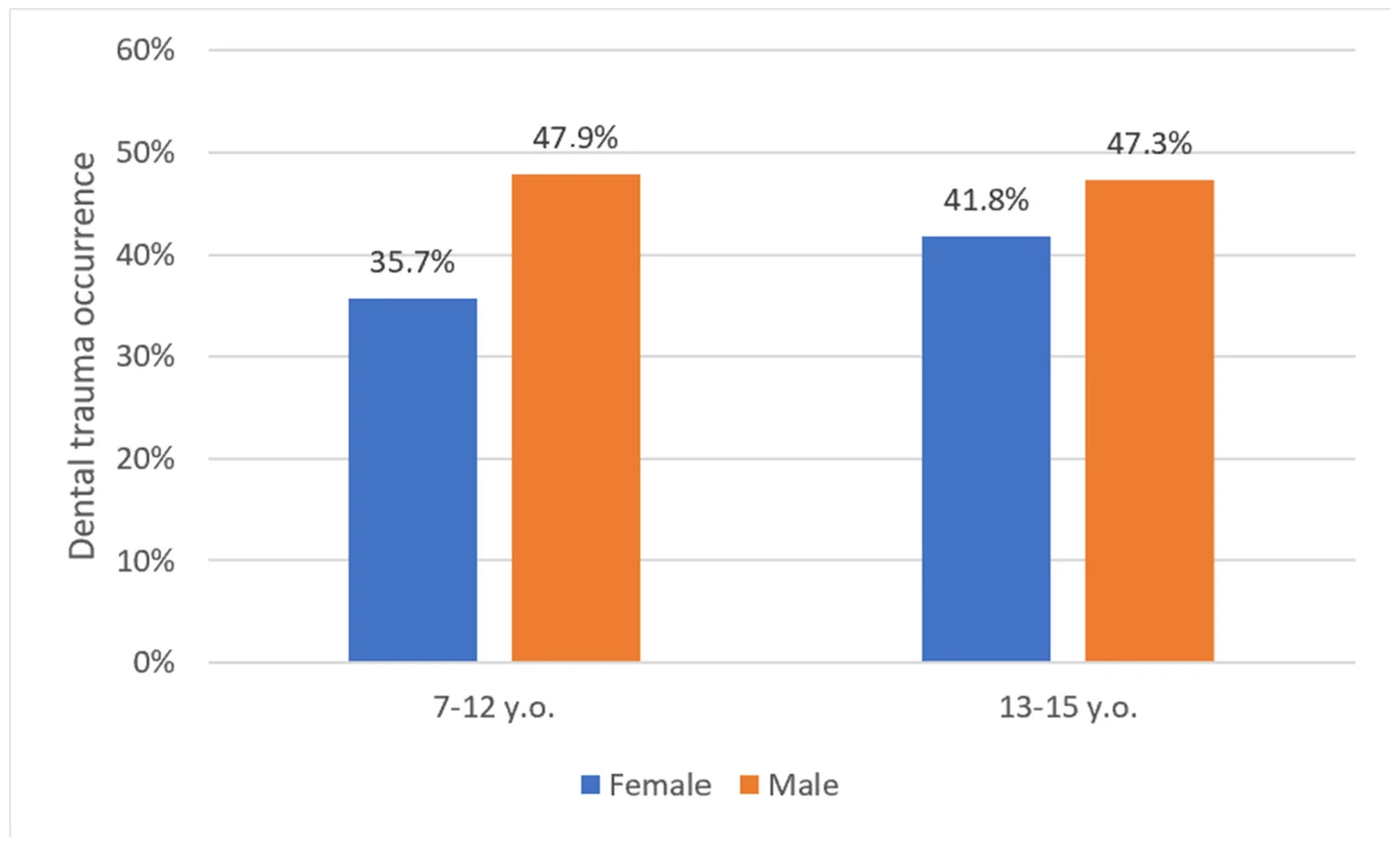
Proportion of children with a reported TDI history according to sex and age group.

The proportion of children with a reported TDI history was higher among boys than among girls in both age groups. The sex-related difference was more pronounced among younger children aged 7–12 years (47.9% vs. 35.7%), whereas among adolescents aged 13–15 years the proportions were more comparable (Figure 1).

### 3.4. Association Between Traumatic Dental Injuries and Sports Participation

The relationship between sports participation and the occurrence of traumatic dental injuries was also evaluated. Chi-square tests or Fisher’s exact tests were performed depending on the distribution of variables and fulfilment of statistical assumptions. Hochberg’s correction was applied to all *p*-values to account for multiple testing across the 13 analysed sport categories.

A reported history of traumatic dental injury was more common among children currently participating in sports than among children who did not report current sports participation (49.7% vs. 31.3%; χ^2^ = 23.79, *p* < 0.001). The observed effect size was low (V = 0.18). Among individual sports disciplines, football was significantly associated with a higher proportion of children with a reported TDI history. A TDI history was reported for 61.8% of children playing football compared with 45.8% of non-football participants (χ^2^ = 8.54, *p* = 0.003; *p_adj_* = 0.036).

In contrast, participation in acrobatics, gymnastics, or dance was associated with a lower proportion of children with a reported TDI history (36.5% vs. 51.8%; χ^2^ = 5.06, *p* = 0.024). However, this result happened to be non-significant after applying Hochberg’s correction (*p_adj_* = 0.264). Therefore, it should be interpreted only as an exploratory result to be reviewed in further research. Similarly, children participating in tennis or table tennis had a significantly lower proportion with a reported trauma history compared with non-participants (9.1% vs. 51.7%; χ^2^ = 15.22, *p* < 0.001; *p_adj_* = 0.001).

No statistically significant associations were found between traumatic dental injuries and participation in basketball, volleyball, swimming, martial arts, athletics, cycling, handball, horseback riding, gym training, or other sports disciplines. The associations between a reported history of traumatic dental injuries and current sports participation are presented in Table 5.

To identify predictors of reported history of traumatic dental injury, a forward stepwise logistic regression analysis was performed using the Wald criterion for variable selection. The candidate predictors were children’s age (7–12 y.o/13–15 y.o), sex (female/male), level of parent’s education (three dichotomous variables—having/not having basic/secondary and higher education level), domicile (five dichotomous variables—living or not living in rural/city < 50,000 inhabitants/city 50–200,000 inhabitants/city 200–500,000 inhabitants/city > 500,000 inhabitants) and current sport participation (yes/no). Independent variables were entered into the model sequentially based on the significance of the Wald statistic.

The final regression model included three variables: participation in sports activities, rural place of residence, and higher parental education level. The model was statistically significant (χ^2^(3) = 48.71, *p* < 0.001), explaining 6.4% of the variance according to Cox and Snell R^2^ and 8.6% according to Nagelkerke R^2^. Other variables (such as children’s age and sex) were not included in the model, as they did not statistically significantly improve the model fit. The model correctly classified 65.7% of all cases, including 78.5% of non-trauma cases and 48.7% of trauma cases.

Among the variables included in the fitted model, current sports participation showed the largest estimated association with a reported TDI history (*OR* = 2.33; 95% *CI*: 1.69–3.21; *p* < 0.001). Rural place of residence was also associated with higher odds of a reported TDI history (*OR* = 1.44; 95% *CI*: 1.02–2.04; *p* = 0.041).

Higher parental education level was associated with lower odds of a reported TDI history (*OR* = 0.52; 95% *CI*: 0.38–0.71; *p* < 0.001). The results of the logistic regression analysis are presented in Table 6.

## 4. Discussion

The present study evaluated the proportion of children with a parent-reported history of traumatic dental injuries and the characteristics of those injuries among children attending a university paediatric dental clinic in the Mazowieckie Voivodeship and assessed their association with current sports participation. A history of traumatic dental injury was reported for 42.9% of the study sample. Current sports participation, male sex, rural residence, and lower parental education level were associated with a higher occurrence of reported TDI history.

The proportion of children with a parent-reported TDI history in the present study was relatively high compared with findings reported in international literature. According to the global meta-analysis conducted by Petti et al. [3], the worldwide prevalence of traumatic dental injuries affecting permanent dentition is estimated at approximately 15%, although considerable differences between populations and study methodologies have been reported. Recent studies among school-aged children demonstrated prevalence rates ranging from 20% to 40% [4,5]. The higher proportion found in the present study may reflect the inclusion of both primary and permanent dentition injuries, high levels of sports participation among children, and differences in socioeconomic and environmental factors.

Current sports participation showed the largest estimated association with a reported TDI history among the variables included in the fitted model. Children currently involved in organised sports had more than twice the odds of a reported TDI history compared with non-participating children. Football participation was also associated with a higher occurrence of reported TDI. These findings are broadly consistent with previous studies indicating that contact and collision sports are associated with dental injuries in children and adolescents [12,13,14,15].

Recent studies continue to identify football and basketball among the sports frequently associated with traumatic dental injuries in paediatric populations [21]. Falls, collisions, and direct facial impacts during physical activity offer plausible explanations for such associations. However, the present study did not establish whether the reported injuries occurred during sports participation and did not assess mouthguard use; these mechanisms therefore cannot be inferred from the present data. Guidelines published by the International Association of Dental Traumatology and the Academy for Sports Dentistry emphasise the importance of properly fitted mouthguards in reducing the incidence and severity of sports-related dental trauma [22]. Recent systematic reviews have also shown that mouthguards reduce the risk of dentoalveolar injuries, particularly crown fractures and avulsions [23]. Educational programmes directed towards children, parents, teachers, and sports coaches may therefore be considered as part of broader dental trauma prevention strategies.

In the present study, a parent-reported history of traumatic dental injury was more common among boys than among girls. This finding is consistent with numerous epidemiological studies reporting a higher prevalence of TDIs among males [8,9]. The greater frequency of injuries observed in boys may be related to higher levels of physical activity, increased participation in contact sports, and more risk-taking behaviours. However, some recent studies suggest that the difference between sexes may gradually decrease due to increasing sports participation among girls and changing lifestyle patterns among children and adolescents [7].

Sociodemographic factors also played an important role in the occurrence of traumatic dental injuries. Children living in rural areas and children of parents with lower educational levels had a significantly higher proportion with a reported TDI history. Similar associations have previously been described in the literature [18,19,24]. Lower parental education may be associated with reduced awareness regarding injury prevention and delayed dental consultation following trauma. Additionally, children living in rural regions may have more limited access to preventive dental care and emergency dental services compared with children residing in large urban centres.

The most common locations of traumatic dental injuries identified in the present study were the home environment and playgrounds or sports fields. These findings are in agreement with previous studies indicating that falls during daily activities and recreational play represent the leading causes of TDIs in children [10,11]. Crown fractures and concussion/subluxation injuries were the most frequently reported injury types, which is also consistent with previous epidemiological reports [16]. In agreement with earlier studies, maxillary incisors were the teeth most commonly affected by trauma because of their anterior anatomical position and greater exposure to direct impacts [8,17].

An important finding of the present study was that nearly half of injured children did not receive first aid within several hours following trauma. Although more than half of respondents reported subsequent dental consultation and regular follow-up appointments, delayed or inadequate emergency management may negatively influence long-term prognosis. Previous studies and international guidelines emphasise that immediate and appropriate intervention following traumatic dental injuries, especially tooth avulsion, is essential for maintaining tooth vitality and improving treatment outcomes [20,22].

Another clinically relevant finding was that 20.3% of parents or legal guardians were unable to identify the specific type of traumatic dental injury. This finding should be interpreted alongside the observation that the home was the most frequently reported location of injury (50.5%) and that 48.9% of injured children did not receive first aid within the first several hours. Together, these findings indicate a potential need to improve caregiver education regarding the recognition of traumatic dental injuries, appropriate immediate first-aid measures, and the importance of timely professional assessment. Practical educational initiatives could include simple guidance on the management of common dental injuries, instructions on appropriate action following tooth displacement or avulsion, and information about when urgent dental consultation is required. Dental professionals may also play an important role by providing caregivers with clear post-trauma instructions and emphasising the need for appropriate follow-up.

The interpretation of the reported injury characteristics requires additional caution. Injuries involving the primary dentition accounted for 57.8% of reported cases, and 29.2% of children with a TDI history were reported to have experienced dental trauma more than once. Given that the study included children aged 7–15 years, with a mean age of 10.6 years, some injuries to primary teeth may have occurred several years before questionnaire completion. This extended recall period may have increased the risk of inaccurate recall and misclassification of the affected tooth or injury type and may partly explain why some caregivers were unable to identify the specific type of trauma. The proportion of children reported to have experienced more than one injury further supports the importance of preventive education and follow-up; however, the cross-sectional design does not allow the causes of repeated injuries or their temporal sequence to be determined.

Given that 18.1% of children with a history of TDI were reported to have experienced an injury at school, preventive strategies should include age-appropriate pupil education, staff training in dental trauma first aid, clear emergency referral procedures, and the use of properly fitted mouthguards during school-based sports associated with a risk of orofacial injury.

These implications should, however, be interpreted cautiously because the study did not establish the circumstances or causes of individual injuries and cannot demonstrate the effectiveness of specific preventive interventions.

The present study has several strengths. The relatively large sample size and the inclusion of clinic attendees residing in both urban and rural areas enabled the assessment of several demographic and behavioural factors associated with parent-reported traumatic dental injuries. However, the diversity of participants’ places of residence should not be interpreted as evidence of population representativeness. Furthermore, the study evaluated the relationship between sports participation and TDIs, which remains insufficiently explored in Polish paediatric populations.

Several limitations should be acknowledged. First, the study was questionnaire-based and relied on parental reporting, which may have introduced recall and misclassification bias. This concern may be particularly relevant to injuries involving the primary dentition, which may have occurred several years before completion of the questionnaire, and to caregiver-reported classification of specific clinical injury types. Second, no clinical examination or review of dental records was performed to verify the reported traumatic dental injuries. Third, participants were recruited using convenience sampling at a single university paediatric dental clinic. Children attending such a centre may differ from the general paediatric population in terms of oral health status, healthcare-seeking behaviour, socioeconomic characteristics, and geographical proximity to the clinic. Consequently, selection bias cannot be excluded, and the observed sample proportion should not be generalised to all children living in the Mazowieckie Voivodeship or Central Poland. In addition, the cross-sectional design does not allow temporal or causal relationships to be established. Moreover, current sports participation was assessed in relation to a retrospective history of dental trauma, and the questionnaire did not establish whether sports participation preceded the injury or whether the injury occurred during sport. The observed associations therefore should not be interpreted as temporal or causal effects of sports participation. Further population-based multicentre studies employing probability sampling and clinical verification of traumatic dental injuries are needed.

In conclusion, a history of traumatic dental injuries was frequently reported in this clinic-based sample of children from the Mazowieckie Voivodeship. These findings provide preliminary regional evidence concerning associations between traumatic dental injuries, sports participation, and selected demographic characteristics, but they should not be interpreted as population-level prevalence estimates.

## 5. Conclusions

A history of traumatic dental injuries was frequently reported among children attending a university paediatric dental clinic in the Mazowieckie Voivodeship. Current sports participation, particularly football, was associated with a higher occurrence of reported dental trauma, while associations were also observed for sex, place of residence, and parental education. The findings support the development of educational initiatives addressing dental trauma prevention and early management among children, parents, teachers, and sports coaches. However, because the study used convenience sampling at a single clinical centre, the results cannot be generalised to the entire paediatric population of the Mazowieckie Voivodeship or Central Poland.

Further multicentre, population-based studies incorporating probability sampling and clinical verification of traumatic dental injuries are needed to establish regional and national prevalence estimates based on clinically verified outcomes.

## Figures and Tables

**Table 1:** Demographic characteristics of the study population.

		*N*	%
Respondent sex	Female	457	62.2
	Male	277	37.7
	No data	1	0.1
Respondent education	Primary	41	5.6
	Secondary	269	36.6
	Higher	421	57.3
	No data	4	0.5
Place of Residence	Rural	184	25
	City: <50,000 inhabitants	101	13.7
	City: 50,000–200,000 inhabitants	192	26.1
	City: 200,000–500,000 inhabitants	50	6.8
	City: >500,000 inhabitants	205	27.9
	No data	3	0.4

**Table 2:** Characteristics of children’s sports activities.

		*N*	%
Playing sports	No	275	37.4
	Yes	459	62.4
	No data	1	0.1
Sport	Football	110	15
	Basketball	40	5.4
	Volleyball	44	6
	Swimming	117	15.9
	Martial arts	90	12.2
	Athletics	33	4.5
	Handball	9	1.2
	Acrobatics, gymnastics, dance	63	8.6
	Tennis/table tennis	22	3
	Cycling	36	4.9
	Gym	5	0.7
	Horseback riding	7	1
	Other	22	3

Note. Children could report more than one sport; therefore, sport-specific percentages do not sum to 100%.

**Table 3:** Characteristics of traumatic dental injuries among children.

		*N*	%
Did the injury occur more than once?	No	217	68.9
	Yes	92	29.2
	No data	6	1.9
Which teeth were injured?	Upper permanent incisors	99	31.4
	Lower permanent incisors	22	7
	Canines	7	2.2
	Premolars	6	1.9
	Molars	9	2.9
	Injury to primary teeth	182	57.8
Where did the injury occur?	School	57	18.1
	Home	159	50.5
	Playground/sports field	114	36.2
	Street	33	10.5
Were you provided first aid for the tooth injury within a few hours?	No	154	48.9
	Yes	159	50.5
	No data	2	0.6
Did you take your child to a dentist after the injury?	No	97	30.8
	Yes	187	59.4
	No data	31	9.8
Type of injury	Concussion, subluxation	92	29.2
	Fracture of the crown	110	34.9
	Displacement/luxation injury	26	8.3
	Intrusive luxation	32	10.2
	Root fracture	24	7.6
	Avulsion	23	7.3
	I don’t know	64	20.3
After the dental injury, did you and your child have regular dental checkups?	No	116	36.8
	Yes	191	60.6
	No data	8	2.5

Note. Percentages are based on children with a reported TDI history (*n* = 315). Multiple responses were permitted for injured teeth, injury location, and injury type; therefore, percentages within these sections may not sum to 100%.

**Table 4:** Association between traumatic dental injuries and demographic variables.

		No		Yes				
Variable		*N*	%	*N*	%	χ^2^	*p*	*Vc*
Gender	Female	210	50.2%	125	39.7%	8.07	0.006	0.10
	Male	208	49.8%	190	60.3%			
Age	7–12 y.o.	316	75.2%	233	74.0%	0.15	0.732	0.01
	13–15 y.o.	104	24.8%	82	26.0%			
						*Z*	*p*	*r*
Parent’s education level	Primary	16	39.0%	25	61.0%	−4.42	<0.001	0.16
	Secondary	132	49.1%	137	50.9%			
	Higher	268	63.7%	153	36.3%			
Place of residence	Rural	92	50.0%	92	50.0%	−2.50	0.013	0.09
	City: <50,000 inhabitants	59	58.4%	42	41.6%			
	City: 50,000–200,000 inhabitants	109	56.8%	83	43.2%			
	City: 200,000–500,000 inhabitants	27	54.0%	23	46.0%			
	City: >500,000 inhabitants	131	63.9%	74	36.1%			

Note: Vc = Cramér’s V; Z = standardized test statistic for the Mann–Whitney U test; r = effect size for the Mann–Whitney U test.

**Table 5:** Association between traumatic dental injuries and sports participation.

		No		Yes					
		*N*	%	*N*	%	χ^2^	*p*	*p_adj_*	*V*
Playing sports	No	189	68.7%	86	31.3%	23.79	<0.001	-	0.18
	Yes	231	50.3%	228	49.7%				
Football	No	189	54.2%	160	45.8%	8.54	0.003	0.036	0.14
	Yes	42	38.2%	68	61.8%				
Basketball	No	215	51.3%	204	48.7%	1.87	0.172	1	0.06
	Yes	16	40.0%	24	60.0%				
Volleyball	No	209	50.4%	206	49.6%	0	0.964	1	0
	Yes	22	50.0%	22	50.0%				
Swimming	No	166	48.5%	176	51.5%	1.72	0.190	1	0.06
	Yes	65	55.6%	52	44.4%				
Martial arts	No	191	51.8%	178	48.2%	1.55	0.213	1	0.06
	Yes	40	44.4%	50	55.6%				
Athletics	No	215	50.5%	211	49.5%	0.05	0.826	1	0.01
	Yes	16	48.5%	17	51.5%				
Handball	No	227	50.4%	223	49.6%	-	0.750	1	0.02
	Yes	4	44.4%	5	55.6%				
Acrobatics, gymnastics, dance	No	191	48.2%	205	51.8%	5.06	0.024	0.264	0.11
	Yes	40	63.5%	23	36.5%				
Tennis/table tennis	No	211	48.3%	226	51.7%	15.22	<0.001	0.001	0.18
	Yes	20	90.9%	2	9.1%				
Cycling	No	215	50.8%	208	49.2%	0.54	0.462	1	0.03
	Yes	16	44.4%	20	55.6%				
Gym	No	228	50.2%	226	49.8%	-	1	1	0.02
	Yes	3	60.0%	2	40.0%				
Horseback riding	No	226	50.0%	226	50.0%	-	0.450	1	0.05
	Yes	5	71.4%	2	28.6%				
Other	No	216	49.4%	221	50.6%	2.95	0.086	0.860	0.08
	Yes	15	68.2%	7	31.8%				

Note. The overall sports participation comparison includes children with available participation and TDI data (*n* = 734). Analyses of individual disciplines are restricted to children who reported current sports participation (*n* = 459); the ‘No’ category therefore means participation in sports other than the discipline shown. Fisher’s exact test was used where expected cell counts were small. V = Cramér’s V.

**Table 6:** Logistic regression analysis of variables associated with a reported history of traumatic dental injuries.

	*B*	*SE*	Wald	*p*	Exp(*B*)	95% *CI*	
						*LL*	*UL*
Higher education level	−0.66	0.16	17.52	<0.001	0.52	0.38	0.71
Rural place of residence	0.36	0.18	4.18	0.041	1.44	1.02	2.04
Playing sports	0.84	0.16	26.54	<0.001	2.33	1.69	3.21
Constant	−0.55	0.16	11.31	0.001	0.58		

Note. Reference categories were no current sports participation, urban residence, and primary or secondary parental education. Exp(*B*) = odds ratio; 95% *CI* = 95% confidence interval; *LL* = lower limit; *UL* = upper limit. *B* = regression coefficient; *SE* = standard error.

## Data Availability

The original contributions presented in this study are included in the article. Further inquiries can be directed to the corresponding author.
